# Whole-blood RNA biomarkers for predicting survival in non-human primates following thoracic radiation

**DOI:** 10.1038/s41598-024-72975-y

**Published:** 2024-10-03

**Authors:** Molykutty J. Aryankalayil, Haaris Patel, Jared M. May, Uma Shankavaram, Michelle A. Bylicky, Shannon Martello, Sunita Chopra, Jim Axtelle, Naresh Menon, C. Norman Coleman

**Affiliations:** 1 Radiation Oncology Branch, Center for Cancer Research, National Cancer Institute, Bethesda, MD USA; 2 ChromoLogic LLC, Monrovia, CA USA; 3 Radiation Research Program, National Cancer Institute, National Institutes of Health, Rockville, MD USA; 4 Radiation Oncology Branch, Center for Cancer Research, National Cancer Institute, 10 Center Drive, Room B3B406, Bethesda, MD 20892 USA

**Keywords:** Non-human primate, Radiation, Radiation bio-dosimetry, Lung radiation, Biomarkers, Respiratory signs and symptoms

## Abstract

Radiation injury, either from radiotherapy or a mass-casualty event requires a health care system that can efficiently allocate resources to patients. We conducted a comprehensive transcriptome analysis of whole blood from a nonhuman primate model that received upper thoracic radiation (9.8–10.7 Gy). Blood samples were collected at multiple time points, extending up to 270 days post-irradiation with a minimum *n* = 6 for initial time points (Day 3-Day 40) and a total number of *n* = 28 primates. No males receiving the higher dose survived to Day 270. Using the Elastic Net model in R we found that pooling biomarkers from Day 3–21 increased our accuracy in discerning survival time, pleural effusion or dose compared to using biomarkers specific to a single day. For survival data, in predicting short term (less than 90 day), medium term (Day 91–269) or long-term survival (Day 270), prediction accuracy using only Day 3 data was 0.14 (95% Confidence Interval (CI) 0.1, 0.19) while pooled data for Male and Female was 0.76 (CI 0.69, 0.82). When pooled data was divided by biological sex, accuracy was 0.7 (CI 0.58, 0.8) for pooled data from Males and 0.84 (CI 0.76, 0.91) for Females. The development of RNA biomarkers as a tool to aid in clinical decision-making could significantly improve patient care in cases of radiation injury, whether from radiotherapy or mass-casualty events. Further validation and clinical translation of these findings could lead to improved patient care and management strategies in cases of radiation exposure.

**Supplementary Information:**

The online version contains supplementary material available at 10.1038/s41598-024-72975-y.

## Introduction

Radiation exposure, either in the small-scale instance of a patient receiving radiotherapy or in a large-scale nuclear disaster, causes damage to normal tissue. Ionizing radiation can pose significant dangers, both in the short-term including acute radiation syndrome (ARS) as well as long-term health effects including increased cancer risk, and the potential for genetic mutations, and organ dysfunction^1,2^. Swiftly assessing the extent of this damage can mitigate long-term dysfunction and efficiently allocate vital medical resources, which will improve patient care and survival^1^. Therefore, the development and utilization of efficient biomarkers are invaluable tools in facilitating swift and accurate diagnoses, ultimately enhancing patient outcomes and preparedness for radiation-related health challenges.

The lungs play a crucial role as a dose-limiting organ in cases of radiation exposure, in nuclear events or radiotherapy, where radiation-induced lung damage significantly influences survival prediction^3–6^. Clinical symptoms of radiation induced lung injury (RILI) and radiation induced pulmonary fibrosis (RIPF) may develop months after radiation therapy in cancer survivors^7^. However, pathogenesis begins in the hours and days after radiation exposure with upregulation of pro-inflammatory cytokines and chemokines from damaged and dying endothelial and epithelial cells, proliferation of fibroblasts and subsequent differentiation into myofibroblasts, and changes to the extracellular matrix^8,9^. The death of endothelial cells leads to occluded blood vessels and vascular damage that result in reduced lung capacity and hypoxia that can lead to respiratory failure and death^10^. While new treatment options such as the immunomodulator Pentoxifylline and the reactive oxygen species scavenger Amifostine have been suggested, the current standard of care is corticosteroid treatment to control symptoms^11^. No methods are currently available which would reverse pulmonary fibrosis, instead research may need to focus on early diagnosis and prevention. Developing effective biomarkers for this purpose could significantly enhance our ability to manage and treat radiation-related lung injuries, ultimately improving patient care and survival in both clinical and nuclear event scenarios.

The current widely accepted method for measuring biological dosimetry typically involves evaluating dicentric chromosome formation in peripheral blood lymphocytes, a time-consuming effort that takes multiple days to process^12^. In mass radiation exposure events, factors like varying dose, multiple exposures, distance from the epicenter, and partial vs. total body exposure complicate the precise determination of physical dose^2^. Because of its accessibility and the wealth of information it contains, whole blood RNA analysis has been used as a diagnostic and prediction tool for diverse human health conditions from cancer to COVID19^13,14^. Studies have shown that exposure to ionizing radiation leads to dose-dependent changes in mRNA expression within whole blood, with certain genes becoming upregulated or downregulated in response to radiation dose^15^. These changes offer valuable insights into the severity of radiation exposure and can serve as potential biomarkers for estimating dose levels. Understanding the dose-dependent mRNA expression profile in whole blood has critical clinical implications, particularly in radiation bio-dosimetry and guiding medical interventions in radiological events^15,16^. Moreover, gene expression studies may enable the development of targeted treatment strategies to mitigate the lasting health impacts of radiation exposure, enhancing public safety, and preparedness for such catastrophic scenarios.

Non-human primate (NHP) models, particularly Macaca mulatta (rhesus macaques), offer significant advantages over small animal models in mirroring human physiology, size, and longevity^17^. They are closest to humans on the evolutionary scale sharing 99.5% sequence similarity in the genome, making M. mulatta a relevant model for molecular and cytogenetic analyses, especially in immune response and signal transduction^18,19^. This emphasizes the NHP, particularly M. mulatta, as a crucial model for advancing our understanding of radiation effects and their potential implications for human health. The doses chosen for this study (9.8, 10.7 Gy) were estimations of the lethal dose to 20% of the population within 180 days of exposure (LD_20/180_) and the LD_75/180_ for upper thoracic radiation^20^. We have previously published on miRNA expression in non-human primate serum and its relationship to clinical findings and cause of death^20^. Work from our lab and others highlights changes in miRNA expression in serum of NHPs after radiation injury, though variability is observed due to underlying genetic differences among individuals^21–23^.

Due to the statistical difficulties associated with analyzing this multitude of time points, some of which contained *n* = 1, we divided the time points into groups. We divided our survival time points into three periods: Day 3–90 (short term), Day 91-Day 269 (medium) and Day 270 (long term) which was when animals were euthanized. Males in the higher dose group died rapidly between Day 60 and Day 90, dropping from 5 animals to 1 survivor. In contrast, lower dose males showed the most precipitous drop in survival between Day 120 and Day 150, dropping from 6 animals to 3, 2 survived until the end of the study. In the higher dose females, 3 out of 6 died between Day 90 and Day 120, while 3 of 6 females who received the lower dose survived until the end of the study. For three of the four study groups, death was not observed until after Day 90.

We obtained blood for 24 time points (Supp Table S1). All animals survived until Day 46^20^. Our goal in this study was to determine a biomarker panel that would help medical personnel triage patients after a mass-casualty event. To this end we tested whether a civilian could arrive at the hospital on a single day and be sorted into a treatment group or if multiple blood draws to determine patient care would be necessary. We also acknowledge that after a mass casualty-event it may take days to weeks to discover and test exposed civilians, so we used a wide time period (Day 3–21) in the hopes of presenting data in the context of potential real-world limitations.

## Results

### Radiation induces gene expression changes that impact immune response and cell survival

**Fig. 1:**
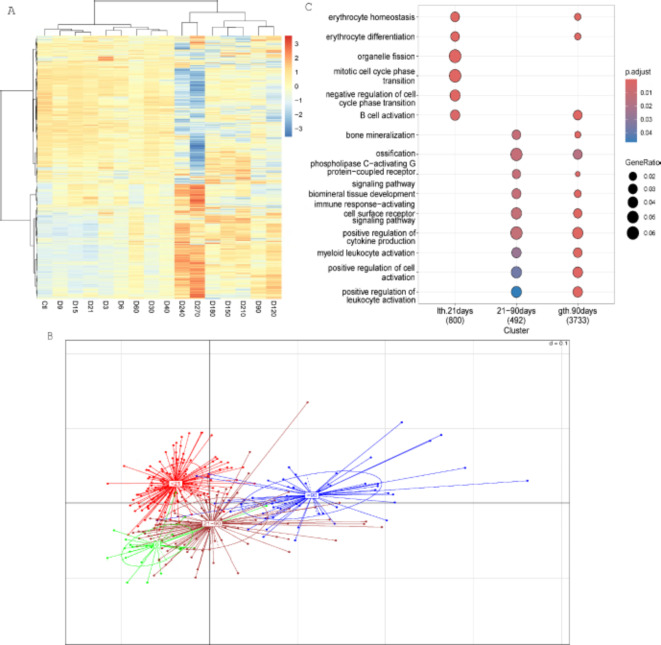
Time course analysis of radiation treated samples. Significant genes (p-value < = 0.05; fold change = > 1.5) were estimated between untreated control and radiation (LD and HD) treated samples. (**A**) Hierarchical cluster analysis with Euclidean distance measure showing log2 transformed normalized data. Mean of the replicate samples was used to represent each time point. Colors represent genes that are up regulated (red) and down regulated with (blue). The legend bar shows the range of the relative intensity data with color scale. Samples were grouped into low (< 21days), medium (21–90 days) and high (> 90days). (**B**) Among groups PCA analysis shows how well the treatment groups separate. The groups are shown as ellipse with colors red (< 21 days), brown (21–90 days), dark blue (> 90 days), and green (untreated control). Each line radiating from the center of the ellipse represents the samples in that group. (**C**) using Enrich GO we compare clusters from clusterProfiler package across the grouped time points. Categories with p-value < 0.05 were chosen for dot plot. The dots are color coded by p-value intensity and the size represent the gene ratio as described in methods. The number in parenthesis under each group label represent number of genes in each group. The abbreviations used in the figure are (lth = less than; gth = greater than).

In Fig. 1A, the heatmap illustrates the alterations of gene expression changes (*p* ≤ 0.05) in whole blood of nonhuman primates over both pre (day − 2) and post radiation exposure time. The heatmap (Fig. 1A) emphasizes distinct patterns of upregulation (red) and downregulation (blue), while yellow indicates a non-significant change. Lower dose (LD, 9.7 Gy) and higher dose (HD, 10.6 Gy) samples were combined and compared to control (-2 day) samples to determine if RNA signatures could be found which differentiated irradiated animals vs. non-irradiated animals at early time points (< 21 days), medium time points (21 < 90 days) and late time points (> 90 days) (Fig. 1B). Data is shown as principal component analysis (PCA) and is used to vitalize the differences among the groups that are made up of samples taken at different time points after radiation or samples from animals that have not yet been irradiated (green). These groups include data from animals prior to radiation (green), samples from animals between 3 and 21 days after radiation (red), samples from animals between 21 and 90 days (brown) after radiation and data from animals 90 days after radiation (dark blue). This separation was chosen based on initial analysis of data where we observed distinct differences in gene expression clusters at these time points. We observed a strong treatment effect based on these gene expression clusters which were evident and could separate unirradiated samples (green) from all irradiated samples. We also observed clusters at distinct time points indicating that gene expression did not remain static after exposure. In total 6882 genes were significantly altered between radiation and control samples for at least one time point. Notably, each primate acted as its own control. Each treatment was compared to the mean of their time point. Gene Ontology (GO) enrichment analysis indicated that these gene expression changes altered diverse biological processes varying from early, medium, and late time points. These processes included B cell activation, erythrocyte homeostasis and differentiation at early and late time points (Fig. 1C). Bone mineralization, ossification and biomineral tissue development occurred at medium and late time points. In addition, more pathway relevant to immune response including cytokine production, myeloid leukocyte activation, and positive regulation of cell and leukocyte activation occurred at medium and late time points.

### mRNA signatures can predict dose received, survival and pleural effusion

Our goal was to determine if we could use mRNA alterations after radiation exposure based on significantly altered genes on each separate day (Day 3, 6, 9, 15, or 21) or if altered genes which were pooled across all days (Day 3–21) would produce the most accurate prediction for dose estimation, survival likelihood, and pleural effusion manifestation. An Elastic Net supervised model was used to select the most important mRNA for each day or pooled days. To display the selected genes in a more meaningful way and to prioritize the important genes, we used Recursive partition tree model. For Dose, data was divided into control (pre-irradiation blood samples, *n* = 24), lower dose and higher dose animals. For Survival, animals were divided into short term survival (survivial ≤ 90 days), medium term survival (91 ≤ survival ≤ 269 days) and long term survival (270 days). Pleural effusion (PE) referred to animals that displayed pleural effusions at any point during necropsy, or non-pleural effusion (NPE), animals who never showed mainifestations of pleural effusion during necropsy.The first recorded death and necropsy occurred on Day 46 in a higher dose male^20^. For survival and pleural effusions the groupings were changed to avoid an unbalanced model. The accuracies and 95% confidence intervals, p-values and important mRNA are shown in Table 1 for Day 3, 6, 9, 15, 21 or pooled data. In addition, we sought to understand how biological sex could impact survival and pleural effusion manifestation and have included pooled data based on biological sex as well.

**Table 1:** Important RNA for predictions of dose, survival and pleural effusion.

Time	Model	Elastic net accuracy (confidence interval)	Elastic net accuracy pvalue	Important RNAs (rank)
Day3	Dose (Ctrl, LD, HD)	0.29 (0.24, 0.35)	1.00E+00	CD300H (23), BLNK (12), ASAH1 (2.1), CALD1 (5.2), CDKN1A (5), CISH (1), CTSB (8.2), RYR1 (14), SLC35E1 (16), AP2S1 (3.2),SLC9A3R2 (3.5), SUCNR1 2.9, TTC8 (2.9), C1QA (1.7), MRC1 (0.9), NDRG2 (0.83), TST (0.89)
Day6		0.4 (0.34, 0.46)	1.00E+00	PLXNA2 (36), LPL (5.1), C1QB (10), C1QC (5), NRDE2(10), HVCN1 (14), PDCD1LG2 (20), ANKRD23 (3.5), AP2S1 (3.5), BRIP1 (3.1), DGKD (0.8), ESRP2 (0.69), FCRL4 (0.92), RAB10 (4.8), RABEP2 (5.7), SOCS2 (3.2), TVP23A (4.7)
Day9		0.38 (0.33, 0.44)	1.00E+00	ADORA2A (0.83), BAP1 (2.6), CCDC130 (2.3), CRY2 (11), CDC27 (0.88), CTDSP1 (4.9), ZNF263 (8.6), CD79A (16), FOXN2 (12), LOC719351 (13), JUN (4.7), GNG7 (0.85), PIGT (3.1), HNRNPA0 (0.76), METTL4 (2.8), RAD23B (2.2), SKIV2L (0.84), TRAM1 (0.81), VPS4A (0.88)
Day 15		0.29 (0.24, 0.35)	1.00E+00	CD79A (13), ACTR3 (0.89), CIAO1 (3), DCAF1 (0.81), DCHS2 (23), IFIT1 (12), KCNH3 (14), SEL1L (7.3), SKIV2L (6.5), SLC48A1 (6.6), SUSD3 (0.85), TMED7 (0.95), UBAC1 (5.2) ALAS2 (9.3), GOLGA5 (4.5), GPX (5.5), MTG2 (4.2), NUDT4 (2.8), PRSS8 (7), RNF115 (4.9), SCYL2 (4.3)
Day 21		0.28 (0.22, 0.33)	1.00E+00	BIN2 (11), C6H5orf52 (3.2), CBX7 (6), CISD2 (5.1), DCAF12 (0.89), DOK1 (3.2), DCUN1D1(3.4), HNRNPAO1 ( IFT172, METTL23 (8.6), NUDCD3 (17), IST1 (4.3), KHDC4 (6.9), R3HDM4 (3.9), RNF115 (0.95), RPS17 (2.1), SAFB (3.4), SPRA (1), VPS72 (7.3)
Pool (day 3-21)		0.77 (0.7, 0.83)	4.20E–29	PLXNA2 (18), ARID5B (1.5), BCL6B (2), CBFA2T3 (1.9) ABLIM1 (3.9), ACKR3 (1.8), EDNRB (22), KLF4 (4.3), LOC106995452 (16), NUDCD3 (20), NUDT4 (3.8), VASH1 (4.1)
Day3	Survival (Ctrl, short term, medium term, long term)	0.14 (0.1, 0.19)	1.00E+00	C1QB (7.5), C1QA (0.96), BAX (3.2), GUCY1B1 (6.6),HTR7 (10), CD68 (0.95), CDKN1A (0.76), C1QC (0.95), KLF4 (6.8), EDA2R (4.1), GNA15 (4.8), LPL (0.79), NPC2 (4.9), NR2C2 (0.86), SELENOP (0.77), TIMP1 (2.2)
Day6		0.32 (0.27, 0.38)	1.00E+00	ABCB1 (6), ARL10 (5.6), ASAH1 (0.89), CKAP2 (7.2), CRY2 (9.5), DCK (0.86), DNAJC6 (0.73), DTL (2), EIF1 (11), FAM219B (9.1), GNA15 (1.9), LOC703641 (12), MZF1 (2.4), NUDT4 (6.1), PUS3 (4.7) TYMS (8.7), JADE2 (12), RNF41 (0.74), UBAC1 (0.74)
Day9		0.38 (0.33, 0.44)	9.90E–01	JADE2 (12),NOP56, PROM2 (12), RBM23(13), ZHX2 (9), AAS (0.89), AGFG1 (0.79), BRCA2 (2.8), CALR (0.8), CRTC2 (0.8), TLR9 (0.72), TRAM1 (0.9), LOC704951 (3.9), MFSD14B (0.88), NOP56 (9.8), RAD23B (0.8), RFK (0.78), RIC8A (3.8), RXRB (0.8), SKIV2L (2.9), SLC2A2 (0.86), STK11IP (2.2), VPS4A (2.2)
Day 15		0.29 (0.23, 0.34)	1.00E+00	SCYL2 (8),UBE2O (8), VWA1 (9.5), BCL2L1 (7.9), DCAF6 (1.5), EIF2AK1 (3.7), FTL (0.59), GPX4 (0.95), HBB (4.8), IGLON5 (2.4), NOC2L (4.7), PAQR9 (6.2), UBAC1 (0.59), SLC48A1 (0.6), RIOK3 (7.4), REXO5 (3.8), R3HDM4 (0.92(, PSMD14 (0,6), PIP4P1 (0.8), PAQR9 (6.2)
Day 21		0.26 (0.21, 0.32)	1.00E+00	DCAF12 (8.1), GANA8 (0.75) HSP90AB1 (10), IRF2BPL (2.7), KDM2A (5.2) LOC100428435 (22), LOC704549 (6.5), RBM23 (11), TAGAP (2.5), THRAP3 (4.2), ZHX2 (9.2), ALAS2 (0.85), ARMCX4 (7.9), ATAD2 (0.86), BRCA2 (3.2), CCz1 (4.7), CEBPB1 (7.1(
Pool (day 3-21)		0.76 (0.69, 0.82)	1.50E–27	AK7 (8.2), APBA2 (2), BCL6B (8.5), DENND2A (7.7), LILRB4 (2.9), HPCAL4 (0.9), KIAA1549 (4.6), LOC106997893 (9.7), LOC114677644 (20), LOC708725 (8,3), LSM12 (3.4), NUDCD3 (18), OAZ1 (6), PROM2 (11)
Pool (male)		0.7 (0.58, 0.8)	6.00E–09	LOC100427190 (16), LOC107000581 (3.4), CPED1 (13), ABHD4 (1.5), ACOT11 (1.5), ALDH3B2 (1.9), BTBD11 (7.3), DENND2A (4.8), FBO32 (5.1)
Pool (female)		0.84 (0.76, 0.91)	4.00E–21	LOC114677644(22), (6.9), NUDCD3 (7.4), GJD3 (5.3), LAMA5 (9.4), ARMCX4 (2.7), AK7 (3.7), AK2 (1.6), ABL2 (2.7), ABCC10 (1)
Day3	Pleural effusion (Ctrl, NPEF, PEF)	0.26 (0.21, 0.32)	1.00E+00	AP2S1 (0.79), ARID58 (2.3), BAX (2.3), BLNK (0.92), C1QB (0.9)CD68 (0.81), DDB2 (4.3), EPHA2 (3), GNA15 (13), LIPA (14), LOC114677063 (6.1), SUCNR1 (13), SYNGR4 (5.1), TEX53 (8), WDR44 (0.77), HTR7 (0.67), NR2C2 (3.8), PAQR7 (3.3) PLLP (6.5) SDC3 (1.9)
Day6		0.42 (0.36,0.48)	1.00E+00	CLEC4C (21), JADE2 (15), LOC114674393 (14), LOC713649 (14), GUCY1B1 (11), LPL (7.3), ANK3 (0.63), BZW3 (4.1) CBLB (0.73), CDC6 (2.8), EI24 (0.24), GNA15 (0.95), LOC703641 (0.85), MGST3 (0.9), MPHOSPH8 (3), MPRIP (4.2), MZF1 (4.7), NPTN (4.6), PBK (2.8), RAD9A (3), RIOK3 (3.4)
Day9		0.54 (0.48, 0.6)	2.30E–02	GATA4 (10), GGA3 (0.83), KLHDC3 (0.88), KLK14 (2.5), PCGF5 (5.2), CRY2 (14), CTDSP1 (1.8), DGKD (0.71), EDA2R (1.4), FAM98C (0.7), FBRSL1 (2.1) SCML2 (14), SLC52A2 (0.92), USP19 (3.6), VPS72 (7.4), WWP1 (6.8), ATP2A3 (0.83), CDC27 (0.94), XYLT1 (0.88)
Day 15		0.37 (0.31, 0.43)	1.00E+00	APEX2 (9.4), LOC100427524 (24), ALAS2 (5.4), BCL2L1 (0.97), C15H9orf78 (0,76), DCUN1D1 (6.5), DDB1 (2.3), DMTN (1.9), FTL (0.96), FXN (0.71), LYZL6 (7.7), RIOK3 (6.3), RNF115 (7.1(, RTCB (0.71), SLC48A1 (0.85)
Day 21		0.34 (0.28, 0.4)	1.00E+00	DRD1 (13), HSP90AB1 (14), IFT172 (10), RAB44 (11), RNF14 (8.6), AP2B1 (0.85), C9H10orf105 (0.9), CBL (0.9), CHD8 (0.84), JAK1 (0.89), LOC705417 (6.6), MARK2 (0.84), PSENEN (0.76), PUM1 (2.5), TMPRSS12 (3.9)
Pool (day 3-day21)		0.89 (0.83, 0.93)	3.10E–24	ABLIM1 (0.89) AK1 (4.7) ATP2A3 (4.7), AUNIP (3.1), LOC706776 (14), CEMIP2 (5.3), CPED1 (3.7), LDLRAP1 (8.1), LOC114676135 (2), LOC701772 (20), LOC712609 (0.94), MFGE8 (7), NOP56 (20), TNFSF14 (2.5)
Pool (male)		0.88 (0.79, 0.94)	9.10E–14	AGAP3 (1.7), BLK (7.8), CD79A (8.8), CEP89 (3.8), FBXO32 (8.3), ZFP28 (18)
Pool (female)		0.85 (0.77, 0.92)	3.60E–13	ADAT (1), APOBEC3H (1.9), ARMCX4 (9.5), LOC100427524 (3.9), LOC701772 (18), MOV10L1 (10), NEK9 (4.8), NUDCD3 (6.4)

Accuracy with confidence intervals, p-value, and important RNAs predicted by Elastic Net for the 3 prediction models used in this study. Data for the Dose model, prediction data for the three-group survival model, and prediction data for the pleural effusion model are shown for Day 3, Day 6, Day 9, Day 15 and Day 21 and pooled data from Day 3-Day21. Tree rank is shown in parenthesis (). Significant results (*p* < 0.05) are observed for Dose and Survival only for pooled data. Predictions for Pleural effusion were only significant after the sexes were separated (male/female) and pooled.

### Separation of sex and dose causes differences in pathway regulation

**Fig. 2:**
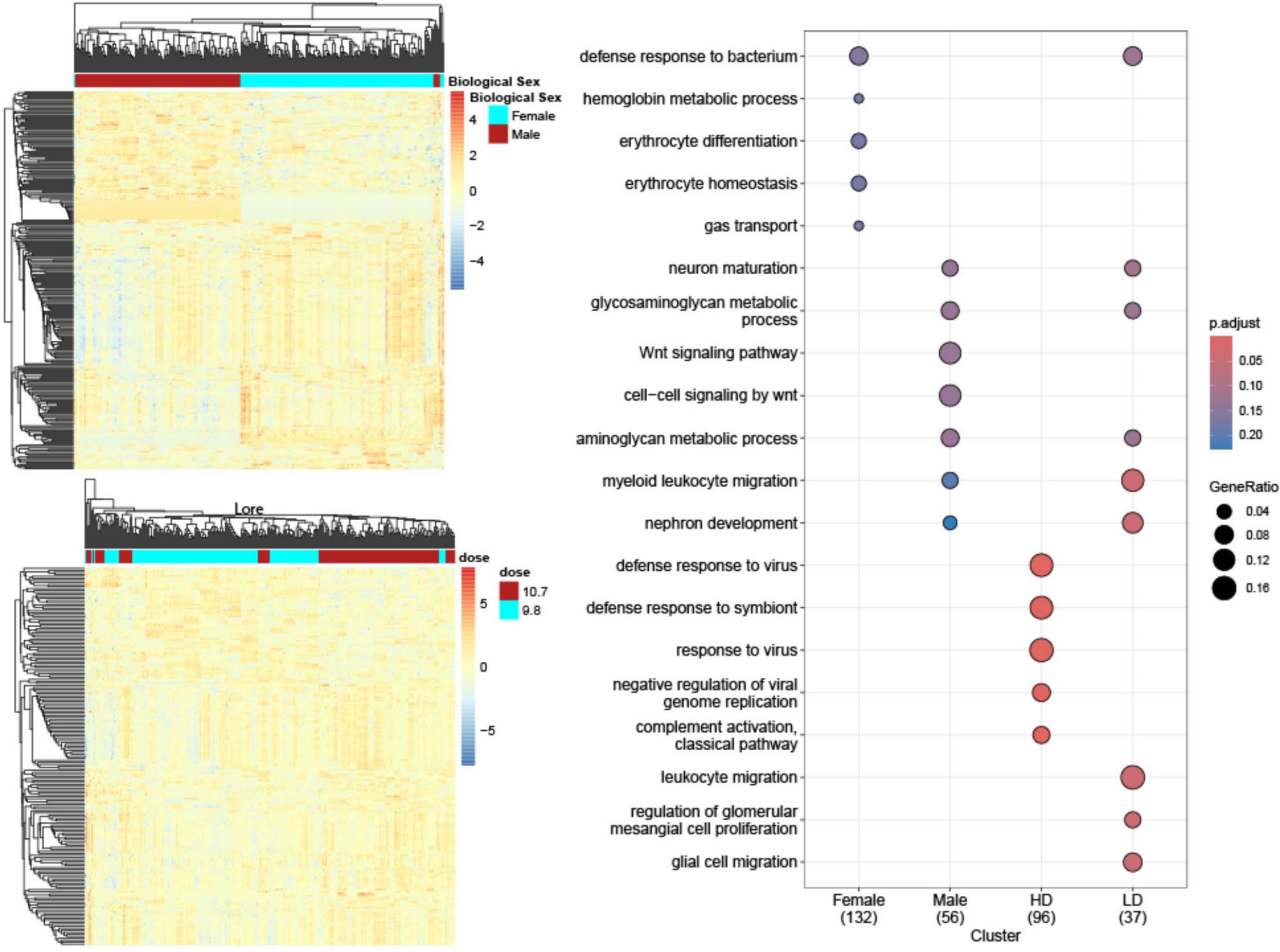
Radiation markers by biological sex and dose. To see the effect of biological sex and dose, significance analysis was performed using the respective groups normalized against untreated same sex controls. Hierarchical cluster analysis using Euclidian distance measure was performed showing the sample grouping by (**A**) biological sex and (**B**) dose. The legend shows the colors used for the sample groups (Red for male, Blue for Female) and the scale bar shows the range of the relative intensity values with colors red representing upregulation and blue indicates down regulation. (**C**) Dot plot of GO biological category compare cluster analysis from clusterProfiler package grouped by biological sex (Male and Female) and dose (HD and LD). The color of dots represents intensity of p-values and size of the dots is relative to gene ratio. Number of significant genes used for each group is shown in parenthesis under each group label.

In Fig. 2, the heatmap illustrates the alterations of gene expression (*p* ≤ 0.05) in whole blood of primates separated by biological sex (male and female) (Fig. 2A), as well as separation of dose (lower dose 9.7 Gy or higher dose 10.6 Gy) (Fig. 2B). Gene ontology (GO) enrichment analysis was performed using these respective groups and normalized against untreated controls (cont.) combining all irradiated samples across all time points (Day 3-270). Categories were chosen based on p-adjusted and gene ratio with the top 5 from each group shown (Male, Female, LD and HD). If pathway changes were predicted in another group, then this was shown as well. For example, Myeloid leukocyte migration was a significantly altered pathway in the LD group, but this pathway also showed alterations in the Male group as well. In female samples we observe 132 altered categories compared to just 56 altered categories in male samples (Fig. 2C). In higher dose samples we observe 96 altered categories compared to just 37 altered categories in the low dose samples. Interestingly we note an upregulation in defense responses to viruses, suggesting an increase in immune expression, in the higher dose samples. In contrast lower dose samples show changes to migration for multiple cell types, (glial, leukocyte, myeloid leukocyte).

### Prediction model of radiation dose

**Fig. 3:**
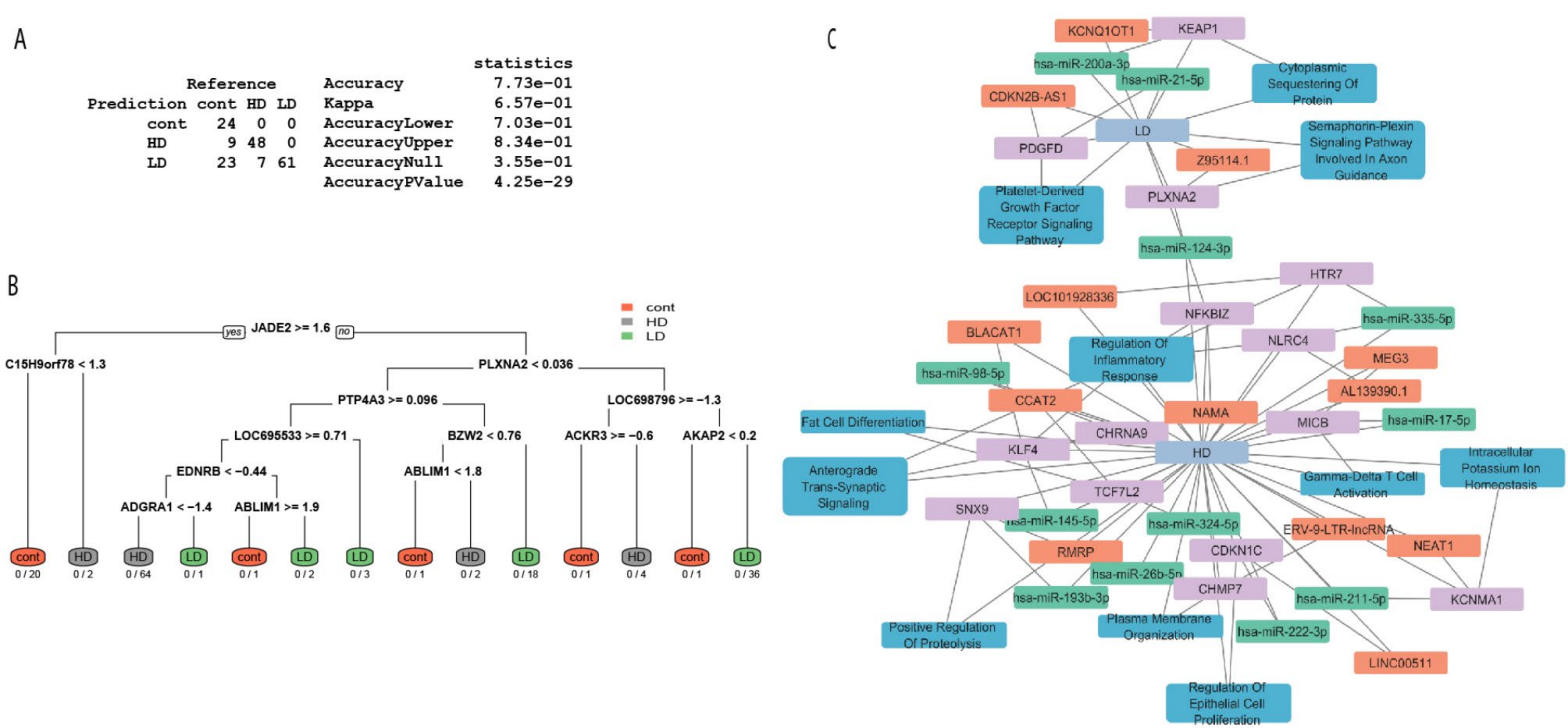
Prediction of radiation dose. Elastic net analysis trained on untreated control and radiation treated samples was performed as described in methods. (**A**) Confusion matrix showing total number of samples per each reference group on x-axis and predicted samples on y-axis. Prediction accuracy statistics are shown for the model. (**B**) CART regression model is used to prioritize and visualize the results of prediction as a decision tree. The groups are color coded and show the terminal nodes. The number of misclassified samples to correctly classified samples after decision rule is indicated under the terminal nodes. (**C**) Using Cytoscape we show a network of pathways and genes (mRNA, miRNA, and lncRNA) associated with low dose (LD) or high dose (HD). Purple indicates mRNA, orange indicates lncRNA, green indicates miRNA and blue indicates pathways of interest. The non-coding RNA (miRNA, lncRNA) shown in this model are predicted based on mRNA expression.

Figure 3 depicts the prediction of radiation dose using a model trained on both untreated control and radiation-treated samples in a timeframe of less than 21 days. Figure 3A offers insights into the model’s performance with a 0.773 (CI 0.703, 0.834) accuracy of estimating radiation doses based on the test data containing time points greater than 21 days, accuracy statistics are also provided. Note: Accuracy Lower is synonymous with the lower 95% confidence interval and Accuracy Upper represents the upper 95% Confidence Interval. Figure 3B displays an additional analytical approach: a recursive partition regression tree model. This model prioritized the most relevant genes for dose prediction and is presented with the probability of predicting each class label, helping us narrow our genes of interest. Using Cytoscape we were able to show the mRNA, predicted lncRNA, miRNA and significant pathways associated with LD and HD radiation (Fig. 3C). We observed PLXNA2 and hsa-miR-124-3p were predicted to be altered in both LD and HD samples. The use of miRNA predictions with human homologs in rhesus macaques due to limitations in macaque annotations is a common approach in translational research, especially when working with non-human primate models.

### Prediction model of survival by biological sex

**Fig. 4:**
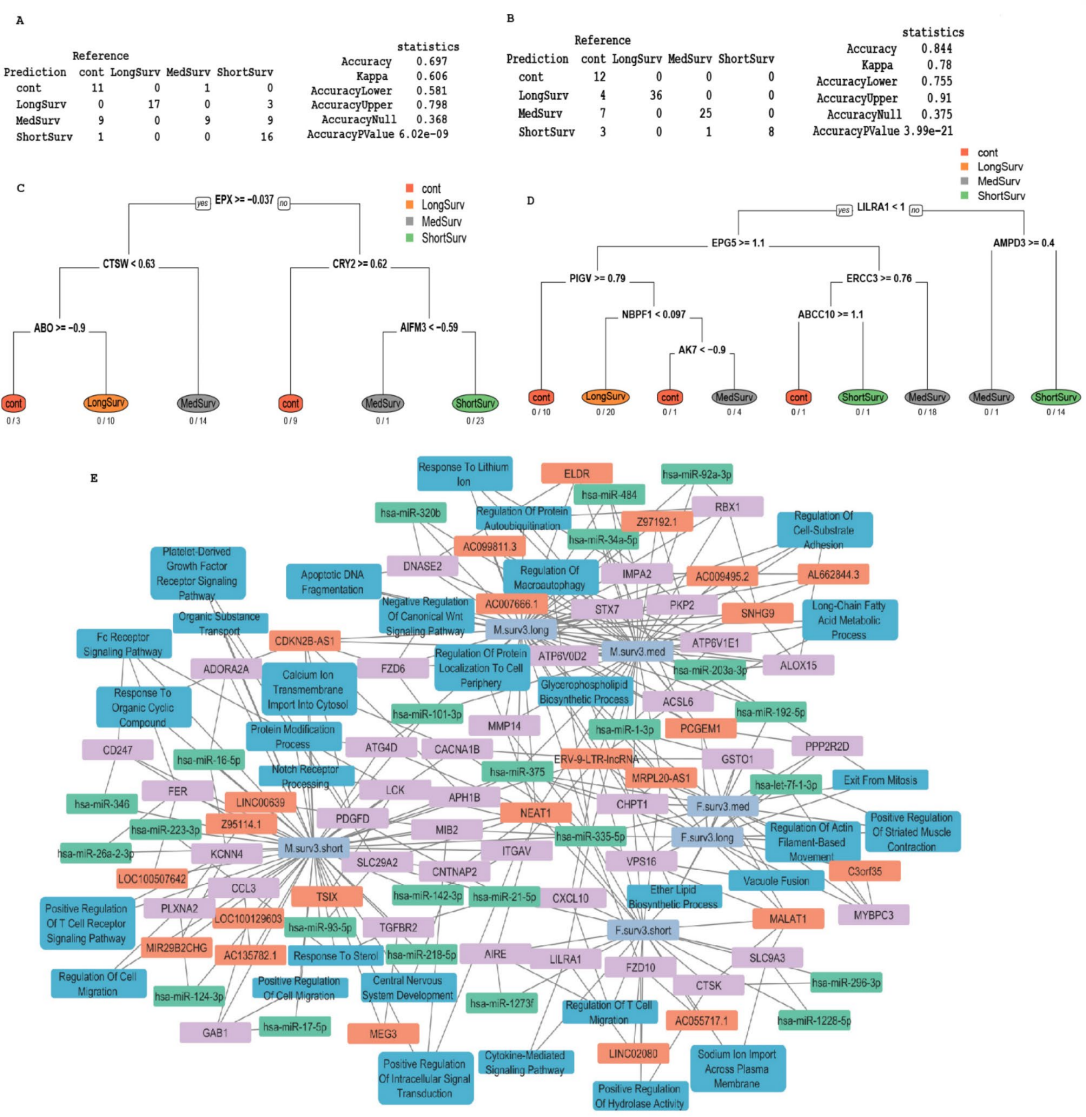
Prediction of survival by biological sex. Genes were selected by significance analysis of samples treated with radiation and stratified by biological sex against same sex untreated controls. Survival time is stratified into short, medium, and long as described in methods. Model evaluated the effect of biological sex on survival before and after radiation. Trained set included samples stratified by biological sex and treated with or without radiation under 21 days followed by prediction on rest of the time points. Results were shown as confusion table and prediction statistics for (**A**) male samples, (**B**) female. Prioritized genes from elastic net model were used to build a decision tree model for (**C**) male and (**D**) female along with number of correct and misclassified samples below each terminal node. (**E**) Using Cytoscape we show a network of pathways and genes (mRNA, miRNA, and lncRNA) associated with short term, and long-term survival for male and female. Purple indicates mRNA, orange indicates lncRNA, green indicates miRNA and blue indicates pathways of interest. For simplicity only male short term and long-term survival and female short term and long-term survival are shown.

Figure 4 presents the prediction of survival outcomes categorized by biological sex using data from Day 3–21. Figure 4A and B display the results of the Elastic Net prediction model for male and female cohorts respectively, accompanied by accuracy scores (male 0.697 (CI 0.581, 0.798) accuracy, female 0.844 (CI 0.755, 0.91) accuracy) to assess the reliability of the predictions in test data. Figure 4C and D display a recursive partition analysis conducted on the genes identified by the Elastic Net model. The resulting predicted tree models are presented separately for males (Fig. 4C) and females (Fig. 4D), showcasing the hierarchical structure of gene interactions and their contribution to survival prediction within each group. Figure 4E depicts the predicted network associated with male short term or long-term survival and female short term and long-term survival, produced in Cytoscape. Interestingly, some genes are sex-specific and are shared across short- and long-term survival such as MALAT1 shared across females, and hsa-miR-101-3p across males. NEAT1 is shared by male short and long term and female short term. Significant genes identified from the Elastic Net model for both male and female (Supplemental Figure S2) cohorts were subjected to an enrichment analysis using Gene Ontology Biological Process (GO BP) ontology. No higher dose males survived until the end of the study, only 2/6 males in the lower dose study survived the full length of the study^20^. In contrast, 3/6 females survived in lower dose and 1/6 in higher dose survived^20^.

### Prediction model of pleural effusion by biological sex

**Fig. 5:**
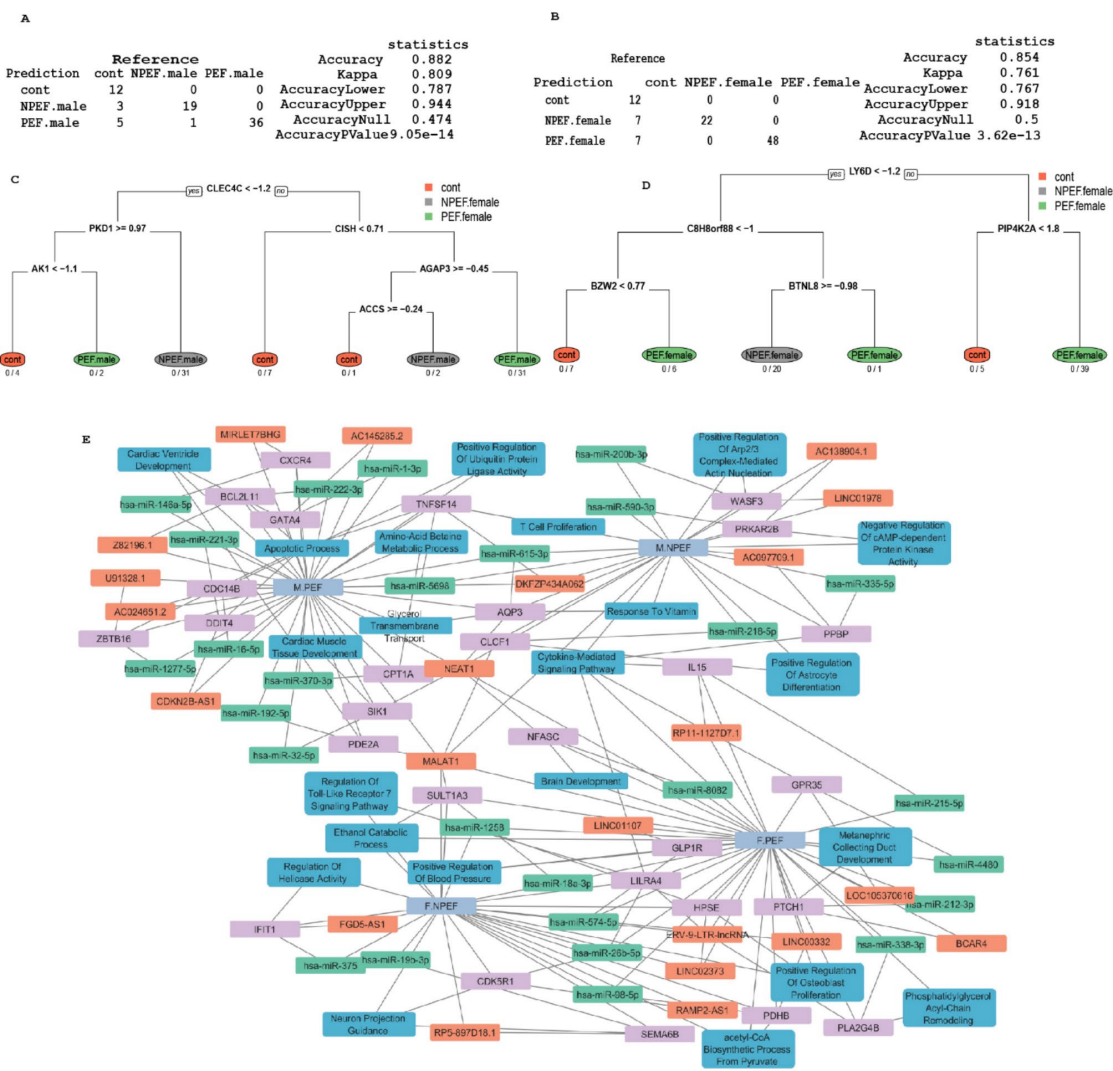
Model evaluation of prediction of pleural effusion by biological sex. Samples are stratified into pleural effusion and further grouped by biological sex as described in methods. (**A**,**B**) Elastic net prediction results and accuracy score of Male (**A**) and Female (**B**). (**C**,**D**) Recursive partition analysis of genes derived from elastic net and shown as predicted tree of (**C**) Male and (**D**) Female. Using Cytoscape we show a network of pathways and genes (mRNA, miRNA, and lncRNA) associated with pleural effusion (PE) or no pleural effusion (NPE) for male and female (**E**). Purple indicates mRNA, orange indicates lncRNA, green indicates miRNA and blue indicates pathways of interest.

In Fig. 5, the analysis focuses on the prediction of pleural effusion categorized by biological sex. Figure 5A and B show the results of the Elastic Net prediction model for male and female cohorts, respectively, along with accuracy scores: male 0.88 (CI 0.79, 0.94) and female 0.85 (CI 0.77, 0.92) on test data to gauge the reliability of the predictions. Pooled data (Day 3–21) was used to predict pleural effusion for animals at any time throughout this study from first euthanasia on Day 46 to end of study on Day 270 and accuracy and important genes are noted in Table 1 (0.89 (CI 0.83, 0.93)). Results for individual animals have previously been published^20^.

Figure 5C and D display a recursive partition analysis conducted on the genes identified by the Elastic Net model. The resulting predicted trees are presented separately for males (5 C) and females (5D), revealing the hierarchical structure of gene interactions and their contribution to predicting pleural effusion within each group. A network schematic was produced in Cytoscape and shows altered genes in PE and NPE samples for male and female. MALAT1 was altered across all four conditions while NEAT1 was altered for both sexes displaying PE and NPE males. Among the predicted microRNA and their gene targets some genes were female specific, appearing for both NPE and PE including: hsa-mir-1258, hsa-mir-26b-5p, hsa-mir-18a-3p and HPSE. TNFSF14, AQP3, hsa-mir-615-3p, hsa-mir-5698 showed altered expression or predicted altered expression in both male samples regardless of outcome (PE or NPE).There were no notable markers shared only with PE male and PE female. Supplemental Fig. S1-3 show bar graphs for important genes relevant for Dose Fig. S1), Survival (Supplemental Fig. S2) and pleural effusion (Supplemental Fig. S3). Supplemental Figure S4 shows validation of mRNA markers chosen based on initial analysis.

## Discussion

We studied the impact of upper thoracic radiation injury (9.8 Gy and 10.7 Gy) on gene expression in whole blood from non-human primates. From this data we were able to predict short, medium, or long-term survival and the manifestation of pleural effusion, demonstrating the utility of gene expression profiling in whole blood as a predictive tool for radiation-induced outcomes. We studied the potential impact of biological sex as a relevant factor in determining biomarkers for pleural effusion and survival. While we saw a trend towards significant improvement when dividing animals by sex for survival prediction where pooled animals showed 0.76 (CI 0.69, 0.82) and females showed 0.84 (CI 0.76, 0.91), this did not rise to the level of statistical significance. Biological sex may have an effect on optimal biomarkers for predicting survival, but further testing of this hypothesis would be needed with a larger cohort. Sex-specific differences in molecular pathway alterations after radiation injury, as shown in Fig. 2, may suggest differences in optimal treatment strategies based on biological sex.

As noted in Fig. 3 heat map, differences between animals that survived to late time points (Day 240 and Day 270) showed the starkest contrast compared to pre-irradiated animals (control). That is, primates’ gene expression did not return to baseline despite surviving but showed increased divergence. This is unsurprising since cardiovascular events, lung and liver dysfunction may occur months to years after radiation injury^24,25^. These late effects of radiation highlight the importance of long-term monitoring and follow-up care for individuals exposed to radiation, particularly those undergoing radiotherapy for cancer treatment or individuals exposed to radiation as a result of nuclear accidents or occupational exposure.

Our gene expression data demonstrating changes to cell division, immune response and hemopoiesis which coincides with other researchers work^26–28^. We observed significant changes to erythrocyte homeostasis and differentiation both in < 21-day samples and in longer term samples (over 90 day). Hematopoietic syndrome, an acute radiation response, occurs when radiation kills hematopoietic stem cells and damages endothelial cells which make up the blood vessels^29,30^. While mature blood cells may survive the initial insult, they will die and replacement cells will not be produced; depending on the radiation dose this may lead to increased risk of infection due to lymphopenia, increased bleeding due to thrombocytopenia, and death. While dysfunction in blood cells and blood vessels is often considered a short-term crisis, here we observe that changes to the hematopoietic system may continue longer term (90 day < ). This can have implications for patient treatment as it is not enough for patients to survive the hematopoietic nadir, long term surveillance may be needed. NHPs who received total body radiation developed spontaneous cerebral lesions up to 13 years post radiation^31^, suggesting greater propensity for spontaneous hemorrhage in radiation exposed individuals.

**Fig. 6:**
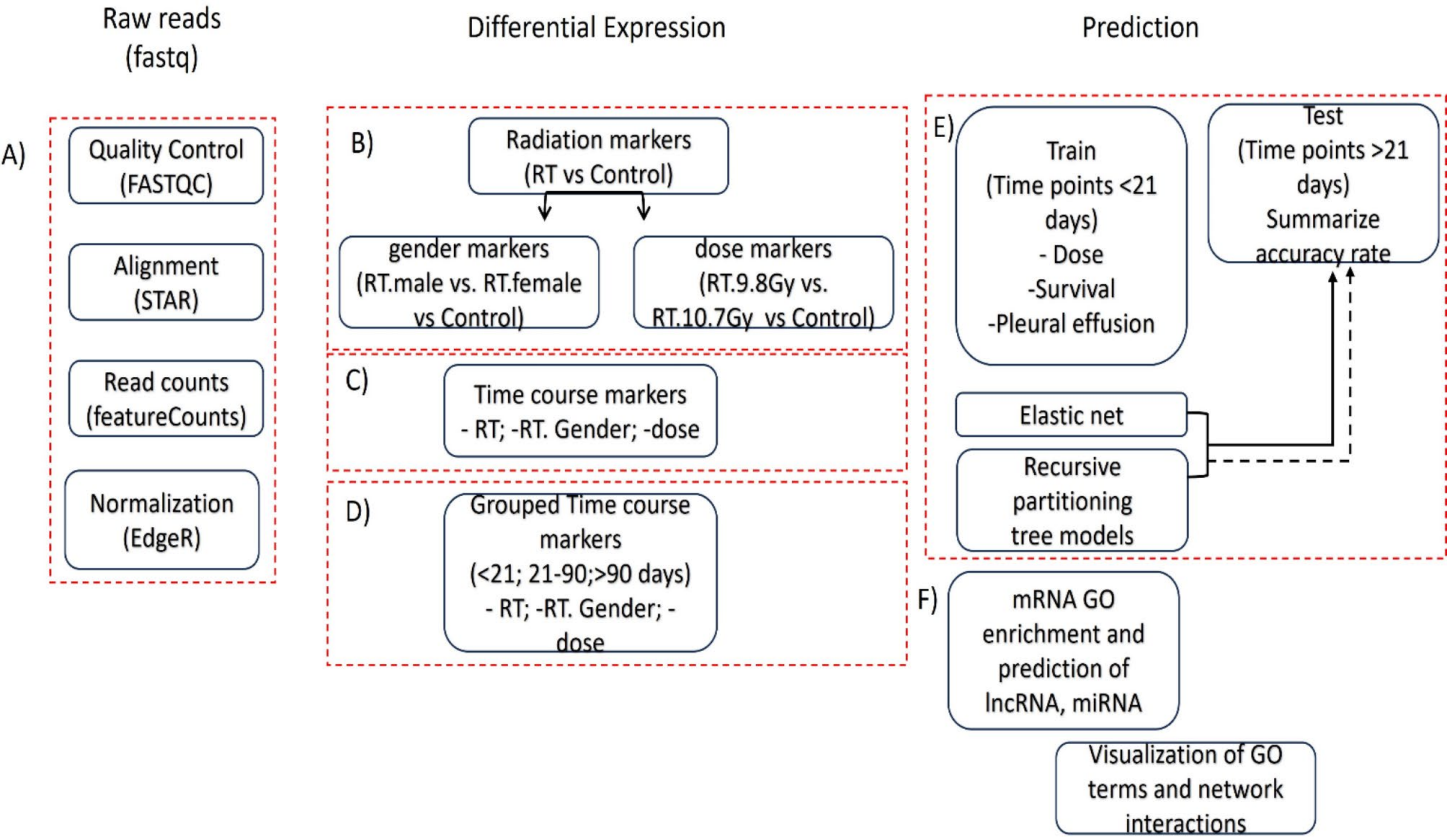
Workflow describes steps of bioinformatics analysis performed in the study. (**A**) processing of raw reads. (**B**) Classification of transcripts performed at 3 levels against untreated control samples: pooled radiation treated samples, biological sex, and dose. (**C**) Time course comparison of the mentioned 3 levels (**D**) time points grouped into 3 major categories as shown. (**E**) prediction of radiation markers using elastic net and regression tree-based methods on 3 major models: (dose, survival time and pleural effusion). (**F**) cross talk between targets and predicted miRNA (and lncRNA) by enrichment analysis.

We further observed immune cell alterations at all time points (Figs. 3, 4 and 6), and B Cell Activation and myeloid leukocyte activation among other immune pathway changes. This B cell activation and proliferation was high in both males and females who developed pleural effusions compared to animals that did not. Interestingly, this follows a different pathway than lung fibrosis which is linked to T cell activation and infiltration^32,33^ into the lung that induces an inflammatory cascade that culminates in myofibroblast activation and deposition. Understanding how different immune cells interact to induce dysfunction is necessary to understand pathogenesis and potential treatment targets. Herein control of B cell activation appears more relevant to preventing pleural effusions in the lung. Prior studies show that radiation induces alterations in immune function, completement cascades, and morphologic changes to lung tissue as we observed^3,18,27^.

We observed more females surviving in the lower and higher dose irradiated groups; however, this was not statistically significant in our experiment, as previously reported^20^. We observed a trend towards greater accuracy in predicting survival but not pleural effusionwhen biological sex was included in the data Despite this, the main pathways altered by radiation were the same for pleural effusion despite biological sex: B cell activation. This redundancy may indicate how important the B cell activation and immune upregulation is to development of pleural effusion. Interestingly, male survival was linked to changes in calcium ion transport and phagocytosis while females showed more changes to cellular organization and cell growth pathways. This calcium ion transport dysfunction may be related to coronary artery calcification, a known side effect of radiation therapy and a risk factor for cardiovascular events^34,35^. We previously noted an increase in damage to the heart after radiation in NHPs^20^. Utilizing RNA panels to predict and discern organ failure prior to clinical manifestation can offer clinicians an opportunity to treat quickly and more effectively. Discovering gene expression markers relevant to injury or death was not simple, in part because of variability in baseline expression within subjects^23,36^. Herein we observe that no singular gene marker can be used effectively to discern radiation dose, predict pleural effusion manifestation, or forecast survival. A panel of biomarkers is necessary for our prediction model.

Our study, while providing valuable insights, has several limitations that need to be acknowledged. First, the low number of animals per group may limit statistical power at specific time points and impacts the overall generalizability of these findings. Secondly, while whole blood is a practical medium for mass clinical testing of civilians, and RNA is a relatively stable biomarker^37,38^, there are concerns about correlating specificity of the potential biomarkers to observed organ damage. More research is needed to determine when RNA changes may correspond to specific organ injury or is while-body response. Furthermore, our study was restricted by limited radiation doses, further studies must include a wider range. Finally, there was an absence of independent animal cohorts to validate algorithms. Follow up studies to test the validity of our biomarkers is necessary.

Other studies have reported higher predictive accuracies and strong validations, such as the study done on GADD45A a biomarker for radiation bio-dosimetry^15^. However, this study used a single mouse strain, which we have previously shown is not even predictive for all mice as in-bred mouse strains show differences in radiation sensitivity^39^. Further, results have shown that mouse strains may show upregulation of a gene while it is downregulated in a human after radiation injury, indicating a potential concern in using them to discover biomarkers^40^. We accept that while our p-values were low, the relative significance of any one gene was minimal when all time points (Day 3–21) were combined. Human cells from different donors have also shown heterogeneity in basal expression and response to radiation^41,42^, this likely represents heterogeneity in the general human population that cannot be ignored. Our work contributes to our theory that no one gene is enough to predict dose, survival or organ injury, rather a panel of genes is needed. Prior studies have used a single miRNA (mir-150) to predict radiation exposure^43^, but more accurate analysis (to predict dose, radiation type, etc.) would require multiple markers^16^.

In summary, our investigation offers crucial insights into the acute transcriptional responses of non-human primate’s post radiation exposure, particularly focusing on mRNA alterations within the initial 21 days following irradiation. By delineating dose-dependent expression patterns and their association with survival outcomes, we have provided valuable initial understandings of the immediate molecular mechanisms triggered by radiation exposure. Overall, our findings contribute valuable insights into the acute transcriptional responses to radiation exposure and sets the stage for further research aimed at improving our understanding of radiation biology and developing effective strategies for mitigating radiation-induced injuries. The identification of pivotal mRNA markers and pathways implicated in the immediate aftermath of radiation exposure sets a solid foundation for future inquiries into therapeutic strategies and biomarker development. Subsequent studies that expand upon our findings, including potential extensions to later time points or investigations into additional biological endpoints, are pivotal for advancing our comprehension of radiation biology and facilitating the development of targeted interventions to alleviate radiation-induced injuries.

## Materials and methods

### Animal selection

In this investigation, both male and female healthy adult Rhesus macaques (M. mulatta, Chinese sub-strain, *n* = 28) with weights ranging from 3.5 to 5 kg during the initial physical examination and ages between 3 and 5 years at the commencement of the study were utilized. The individual housing conditions adhered to the standards outlined in the Animal Welfare Act and recommendations provided in the Guide for the Care and Use of Laboratory Animals (National Research Council 2011). The animals were kept in a controlled environment, maintaining a temperature between 18 and 29 °C and a relative humidity between 30 and 70%. Their diet consisted of PMI LabDiet^®^ Fiber-Plus^®^ Monkey Diet 5049 biscuits, administered twice daily, with fasting before irradiation and examinations. Supplementary fruits, vegetables, and dietary supplements were incorporated throughout the study. Fresh drinking water was available ad libitum. Regular monitoring of both the diet and drinking water ensured the absence of contaminants that could influence the study outcomes. The study was conducted in a laboratory approved by the Association for Assessment and Accreditation of Laboratory Animal Care (AAALAC), with approval from the Institutional Animal Care and Use Committee. SNBL USA possesses an Animal Welfare Assurance issued by the Office of Laboratory Animal Welfare (OLAW) and is registered with the United States Department of Agriculture. The research adheres to the guidelines of the Animal Welfare Act and the recommendations in the Guide for the Care and Use of Laboratory Animals (National Research Council 2011). Additionally, the study is reported in accordance with ARRIVE guidelines.

### Irradiation procedure

Animals were allocated to treatment groups using a stratified randomization scheme based on body weight. A 14-day quarantine and acclimation period in the study room preceded photon beam irradiation. The irradiation process was meticulously supervised by a Medical Physicist, with quality assurance conducted in the morning before the procedure. Utilizing a Varian CLINIC 21EX linear accelerator with a maximum of 6 MV, Group 1 consisted of three male and three female non-human primates (NHPs), while Group 2 included four males and four females. Group 1 received a total dose of 9.8 Gy, delivered to the thorax at a rate of 600 ± 10 cGy/min, and Group 2 received a total dose of 10.7 Gy at the same rate. These doses were designed to emulate LD20/180 and LD75/180, respectively. Radiation treatment planning involved thorax CT scans (200 kV potential, 110 mAs, 1.5 rotation time, 1.0 pitch level, B70s kernel, and 1.5 mm slice thickness) and Varian Eclipse TPS v13.6 software. Prior to irradiation, animals fasted for at least 12 h, and an antiemetic (Ondansetron HCl, 1.0 mg/kg) was administered between 15 and 90 min before the procedure. Anesthesia with ketamine/xylazine preceded transportation to the irradiator, where sedated animals were positioned supine, head to gantry, arms overhead, with the gantry at 180 degrees. Imaging verification ensured proper positioning, and the dose was administered with approximately 50% contribution from both anterior–posterior and posterior-anterior beams.

### Veterinary care in irradiated rhesus macaques

Throughout the study, vigilant veterinary care and analgesic management were integral components of the animal welfare protocol for irradiated Rhesus macaques. Cage-side clinical observations occurred twice daily, with a minimum 6-hour interval between assessments. Veterinary physical exams, conducted during acclimation and every 30 ± 3 days during sedation for CT scans, encompassed evaluations of body condition, hydration, capillary refill time, rectal temperature, heart rate, respiratory rate, and organ system function. Non-sedated measurements of pulse oximetry, rectal temperature, and respiratory rates were carried out twice weekly, once weekly, and every 3 days, respectively. All animals received oral tramadol (1–4 mg/kg) or buprenorphine (0.01 mg/kg) twice daily from post-irradiation Day 4 to Day 25. Tramadol was the primary medication, mixed with food or treats as needed. In cases of oral administration refusal for two consecutive treatments, animals were switched to buprenorphine for the remaining treatment period. Animals experiencing distress after tramadol or buprenorphine received 0.02 mg/kg of buprenorphine twice daily. Tachypnea indicative of pneumonitis (non-sedated respiratory rate ≥ 80 breaths per minute) triggered corticosteroid treatment. For the initial episode, dexamethasone was administered IM in a tapered regimen. A second episode within 7 days of stopping the first course received an alternate dexamethasone regimen. Animals completing two courses of corticosteroid treatment with an additional non-responsive tachypnea episode met euthanasia criteria. This stringent veterinary care protocol aimed to ensure the well-being and responsiveness of the macaques during and after irradiation.

Euthanasia decisions were substantiated through veterinary examinations, hinging on the presence of either a “single criterion” or a combination of two or more “combination criteria.” Singular criteria encompassed enduring pain or distress despite the administration of two consecutive increased doses of buprenorphine (0.02 mg/kg IM twice daily), immobility exceeding 15 min, respiratory distress characterized by labored breathing, open-mouth breathing, a respiratory rate exceeding 80 breaths per minute with unresponsiveness to treatment, uncontrolled hemorrhage from any orifice, or indications of severe dehydration, evidenced by skin tent time exceeding 3 s, sunken eyes, rapid and weak pulse, cold extremities, and/or a comatose state. Combination criteria included a weight loss surpassing 25% of baseline for two consecutive days, severe injury, hyperthermia (rectal temperature exceeding 41 °C), hypothermia (rectal temperature below 35 °C), or complete anorexia for 48 h.

Severity scores for lung and heart conditions were assessed during necropsy based on specific criteria. For the heart, severity scoring incorporated evidence of heart lesions, fibroplasia in the interstitium, myocardial degeneration, mononuclear cell infiltration, hemorrhaging, fibroplasia of epicardium, and fibrin deposition in the interstitium. In the case of the lungs, severity assessment involved evidence of fibroplasia in the interstitium, macrophage infiltration into alveolar bronchioles, fibroplasia in the pleura, edema in alveolar spaces, mononuclear cell infiltration in the interstitium, bronchiole hyperplasia, fibrin deposition in the alveolar space, hemorrhage in alveolar space, presence of mononucleated macrophages, and infiltration of mixed inflammatory cells.

### Blood collection procedure

Approximately 3.5 mL of blood was obtained from the peripheral vein of conscious animals. The site underwent disinfection using an alcohol swab, and a lancet was firmly pressed against the skin to lance it. The initial blood drop was wiped with an alcohol swab and then collected into an RNA protect Animal Blood Tubes (Cat # 76554). Subsequently, the samples were shipped to the National Cancer Institute (Bethesda, MD) on dry ice.

Blood samples were procured from each of the animals (*n* = 28) two days before irradiation as control, every three days post-irradiation for the initial 30 days, on days 40, 50, and 60, and approximately every 30 days until the euthanization of all surviving animals at day 270 post-irradiation. Animals displaying signs of severe acute radiation syndrome between scheduled blood collection points were euthanized, and a final sample was drawn. For animals manifesting severe acute radiation syndrome during scheduled collection intervals, they were slated for terminal necropsy, and a concluding blood sample was obtained. Number of animals included for each time point is included as a supplementary table (Supp table S1).

### RNA isolation

Total RNA was isolated using the RNeasy Protect Animal Blood Kit (QIAGEN) Cat # 73,224 from the whole blood without separating serum or plasma. Briefly, all blood cells are lysed in the RNA Protect Animal Blood tubes (QIAGEN) and RNA (intracellular and extracellular) was precipitated along with cell debris. The pellet was washed once with RNase free water. RNA isolation was subsequently performed from the pellet using the manufacturer’s protocol. Total RNA obtained was further purified and concentrated using the RNA Clean-up and Concentration kit (Cat # 61000, Norgen Biotek Corp., Thorold, Canada). Quality and quantity of the RNA samples were assessed using an Agilent Bioanalyzer with the RNA6000 Nano Lab Chip (Agilent Technologies; Santa Clara, CA). The RNA integrity numbers were consistent for all the samples and ranged from 8 to 9. Samples were sent for processing to Novogene.

### Novogene sequencing

Isolated RNA sample quality was assessed by Fragment Analyzer High Sensitivity RNA Assay (Agilent Technologies Inc., California, USA)] and quantified by Invitrogen BR RNA Qubit (ThermoFisher, Massachusetts, USA). Ribosomal RNA depletion was performed with Ribo-Zero Plus rRNA Removal Kit (Illumina Inc., California, USA). Subsequently, library was constructed by using Truseq Stranded Total RNA kit (Illumina, California, USA). Final libraries quantity was assessed by Qubit 2.0 (ThermoFisher, Massachusetts, USA) and quality was assessed by TapeStation HSD1000 ScreenTape (Agilent Technologies Inc., California, USA). Average final library size was about 400 bp with an insert size of about 250 bp. Illumina^®^ 8-nt dual-indices were used. Equimolar pooling of libraries was performed based on QC values and sequenced on an Illumina^®^ NovaSeq 6000 (Illumina, California, USA) with a read length configuration of 150 PE 40 M PE reads per sample (40 M in each direction).

### Data analysis

Methods of data analysis are shown in a schematic (Fig. 1). To assess the sequencing quality, FastQC was performed after adapter sequence trimming. After confirming all sequences passed the QC score of greater than 30, sequences were subjected to the pipeline as described in Fig. 1. In short, The FASTQ sequences were trimmed using CutAdapt v. 2.10 [10.14806/ej.17.1.200] with the following settings: “--nextseq-trim = 2 --trim-n -n 5 -O 5 -q 10,10 -m 15 -j 0” and aligned using STAR v. 2.7.6a [10.1093/bioinformatics/bts635] with parameters set as recommended by ENCODE3 consortium (https://www.encodeproject.org/pipelines/ENCPL444CYA/). Macaca mulatta (Rhesus monkey) genome assembly Mmul_10 (release date: Feb. 2019) with RefSeq annotation (accession number GCF_003339765.1) was used as a reference. Counting was performed with the help of Subread package v. 2.0.1 [10.1093/bioinformatics/btt656]. The count matrix was processed in R^44^ for further analysis.

Data was pre-processed by removing low noise level counts using default settings (keep read counts minimum value of 3 in atleast 80% of samples) and normalized by upper quartile method from edgeR package. The ability to measure gene expression on genome scale is a promising achievement in molecular biology. However, even with improved statistical methods many of the problems associated with microarray technologies such as unwanted sources of variability is still an issue. The guanine-cytosine content (GC-content) has a strong sample-specific effect on gene expression measurements that, if left uncorrected, leads to false positives in downstream results. We have tested both TMM and quantile normalization most commonly used methods for calculating normalization factors. We find that Quantile is the more robust of the two, as it normalizes for nonlinear effects as well as simple scaling. Conditional quantile normalization algorithm combines robust generalized regression to remove systematic bias introduced by deterministic features such as GC-content and quantile normalization to correct for global distortions^45^. Data is transformed into log2 scale for further analysis. Data quality was assessed before and after normalization. Differential gene expression was calculated by Student’s T-test followed by false discovery rate (FDR) estimation by Benjamini Hochberg method^46^. For Time course analysis, pairwise comparison with radiation treated and untreated control by T-test followed by Dunnett’s test^47^. Differences in male vs. female was calculated using same sex untreated control samples. For prediction of radiation sensitivity on dose, radiation and sex differences on survival, and pleural effusion data was processed first to select optimal features for a given comparison using Boruta algorithm in R^48^ followed by elastic net analysis^49^. The accuracy scores for each model were generated after 5-fold cross validation and represented with 95% upper and lower confidence interval limits. Basically, an accuracy that falls between 95% confidence interval indicate that the probability of prediction is correct 95 out of 100 times. The elastic net is a powerful machine learning algorithm that performs both variable selection and regularization simultaneously. It is a regularized regression technique that is used to deal with the problems of multicollinearity and overfitting, which are common in high-dimensional datasets. This algorithm works by adding a penalty term to the standard least-squares objective function making it more robust. Followed by elastic net, to address if individual treatment can be classified from pooled data and generate easy to visualize decision rules, we used predictive classification and regression trees (CART)^50^ from rpart (Recursive Partitioning And Regression Trees) package in R. The algorithm of decision tree models works by repeatedly partitioning the data into multiple sub-spaces, so that the outcome in each final sub-space is as homogeneous as possible. This approach is technically called recursive partitioning. The produced result consists of a set of rules used for predicting the categorical variable, for classification trees, and the decision rules generated by the CART predictive model are visualized as a binary tree. The data was split to training (< 21days) and test (> 21days) for model estimation and validation.

### Association of miRNA and Long noncoding RNA

To check potential lncRNA-mRNA-miRNA interactions, NcPath tool^51^ was employed. We downloaded experimentally verified lncRNA-mRNA and the interaction relationships of miRNAs and their target genes were downloaded from 8 distinct databases. We mapped significant mRNAs from our study to genes associated with lncRNA-mRNA and miRNA-mRNA lists and kept only experimentally validated targets.

### Enrichment and network analysis

Gene ontology enrichment analysis was performed on significant genes using clusterProfiler package in R^52^. Gene ratio is estimated (number of significant genes belonging to the pathway/ total number of genes in the pathway) for each cluster. Interaction networks using the Cytoscape 3.9.0 program (https://cytoscape.org/).

### Real time RT-PCR analysis of RNAs

200 nanograms RNA was reverse transcribed using High-Capacity cDNA Reverse Transcription Kit, Applied Biosystems (Cat # 4368814) according to the manufacturer’s protocol. qRT-PCR was performed using Applied Biosystems™ TaqMan™ Fast Advanced Master Mix for qPCR (Cat #4444557) for a select set of genes from Applied Biosystems. The following RNA primers were purchased from Thermofisher and were used (DCAF12 (Rh02869704_m1)), ACTG2 (Rh02800953_m1), LPL (Rh02930626_m1), DCUN1D1 (Rh02925260_mH), CXCL10 (C1QB (Rh00608019_m1), C15H9orf78 (Rh00535105_m1), JADE2 (Rh02869323_m1)). PCR reactions were performed in ABI QuantStudio. with GAPDH (Applied Biosystems, Rh 02621745) being used as the normalizing gene. Fold change values were calculated as previously reported from our lab^53^.

## Electronic supplementary material

Below is the link to the electronic supplementary material.

Supplementary Material 1

Supplementary Material 2

Supplementary Material 3

## Data Availability

Data will be available in the NCBI GEO database submission #GSE264593.
