# Supplementary material for: Whole-blood RNA biomarkers for predicting survival in non-human primates following thoracic radiation
Source: Sci Rep. 2024 Oct 3;14:22957. doi: 10.1038/s41598-024-72975-y (PMC11449919; doi:10.1038/s41598-024-72975-y)
Supplement: Supplementary file 1 — Supplementary Material 1 [file 41598_2024_72975_MOESM1_ESM.pptx]

## Slide 1
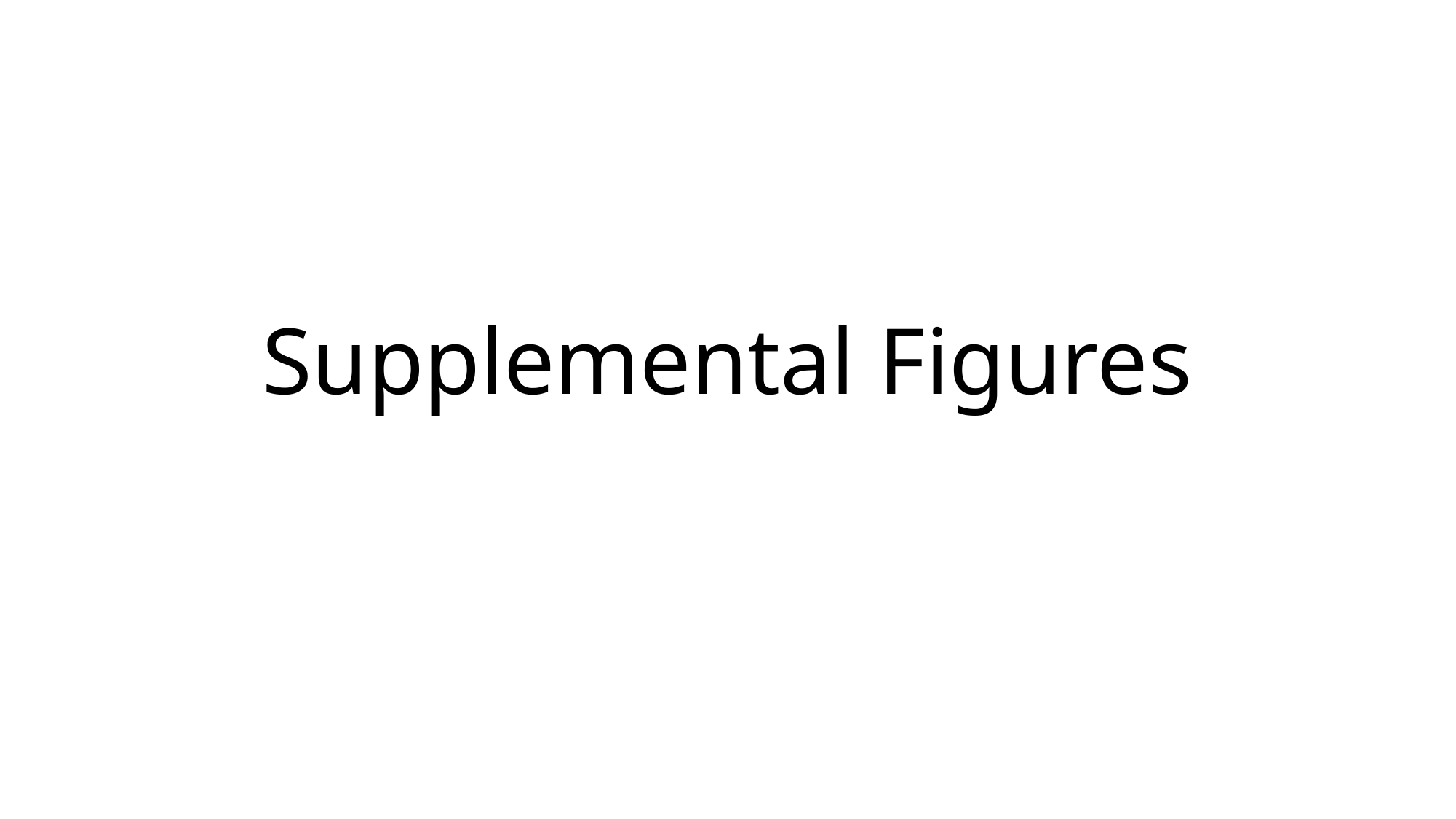

# Supplemental Figures

## Slide 2
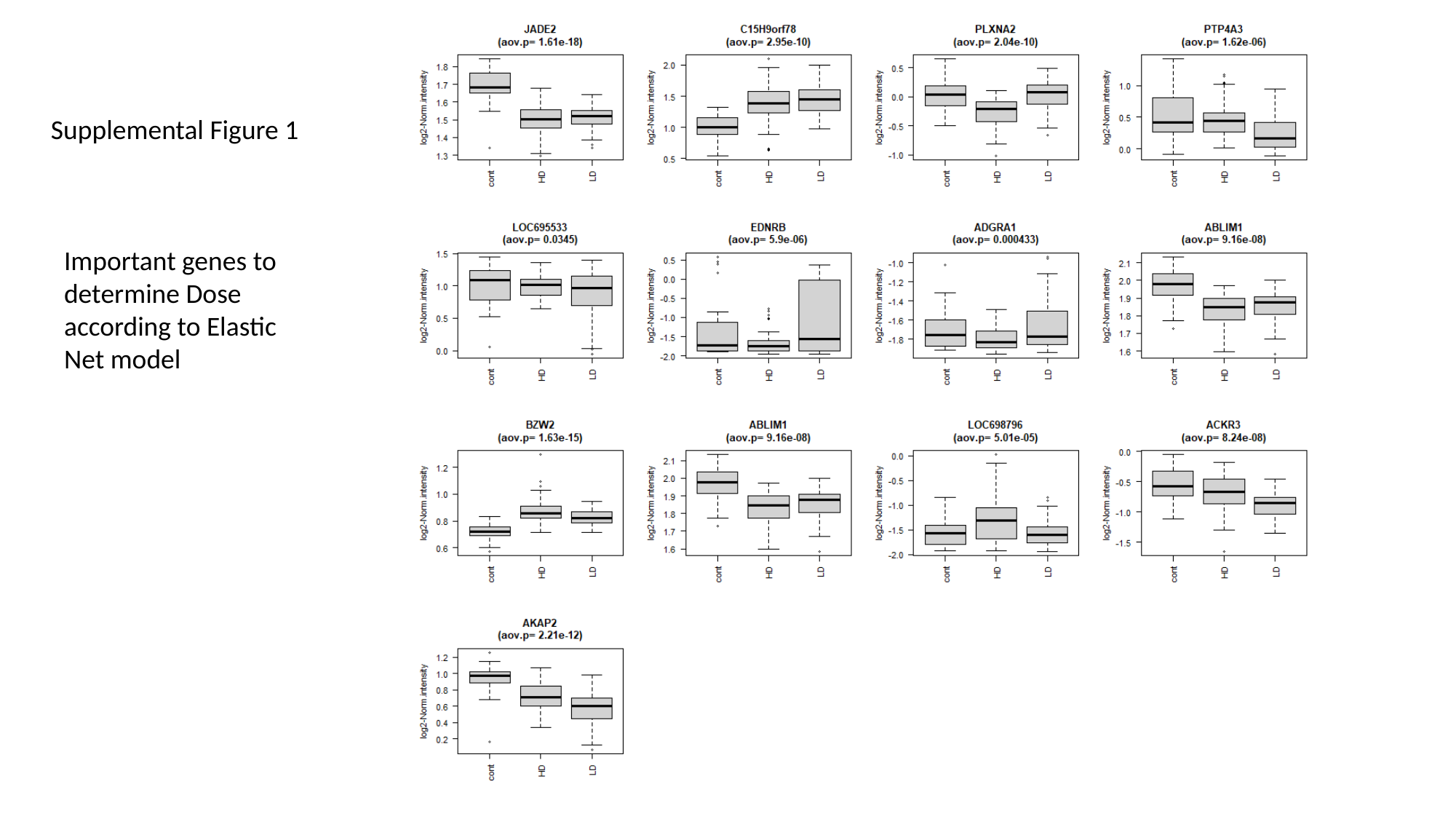

Supplemental Figure 1
Important genes to determine Dose according to Elastic Net model

## Slide 3
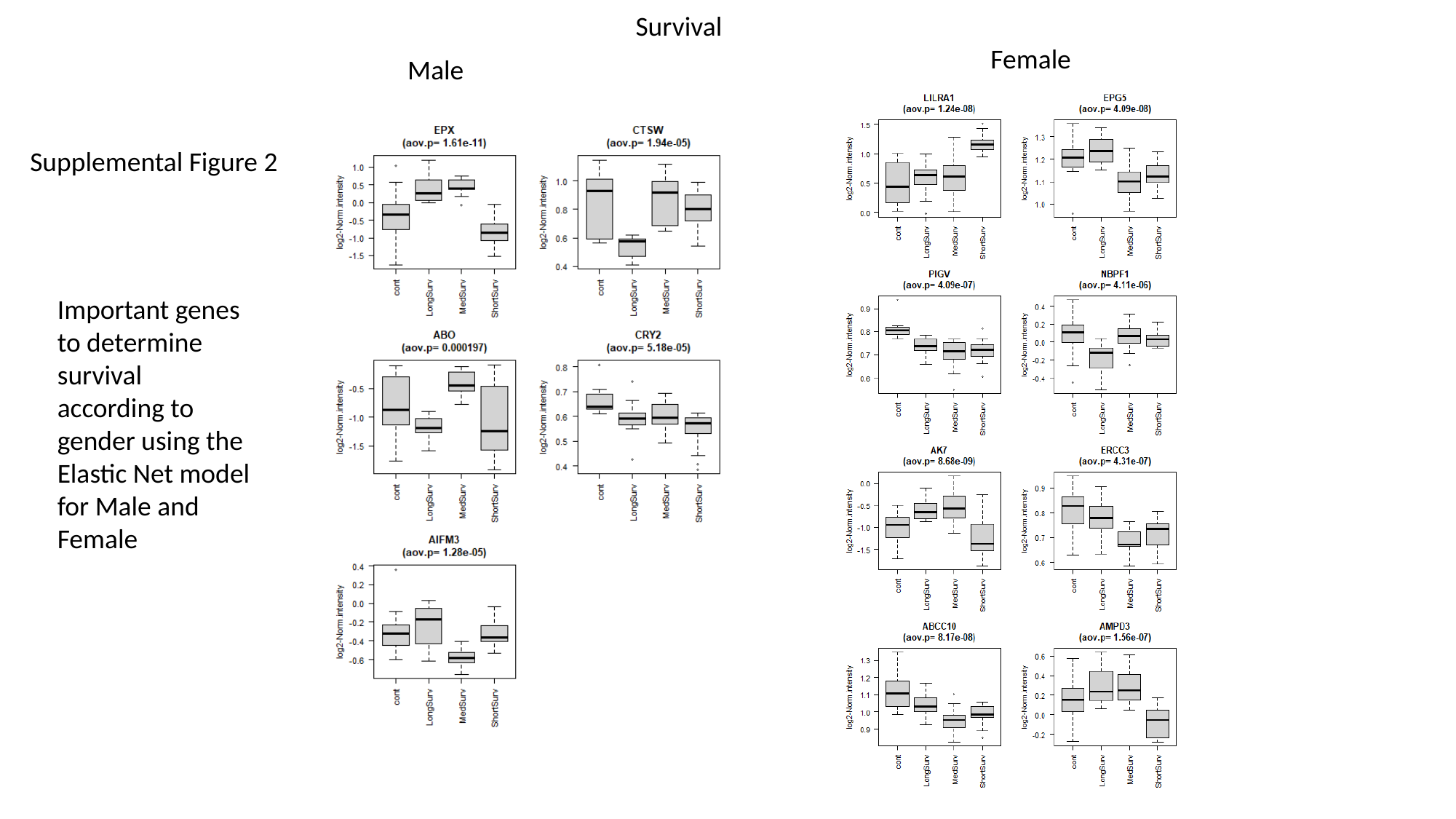

Survival
Female
Male
Supplemental Figure 2
Important genes to determine survival according to gender using the Elastic Net model for Male and Female

## Slide 4
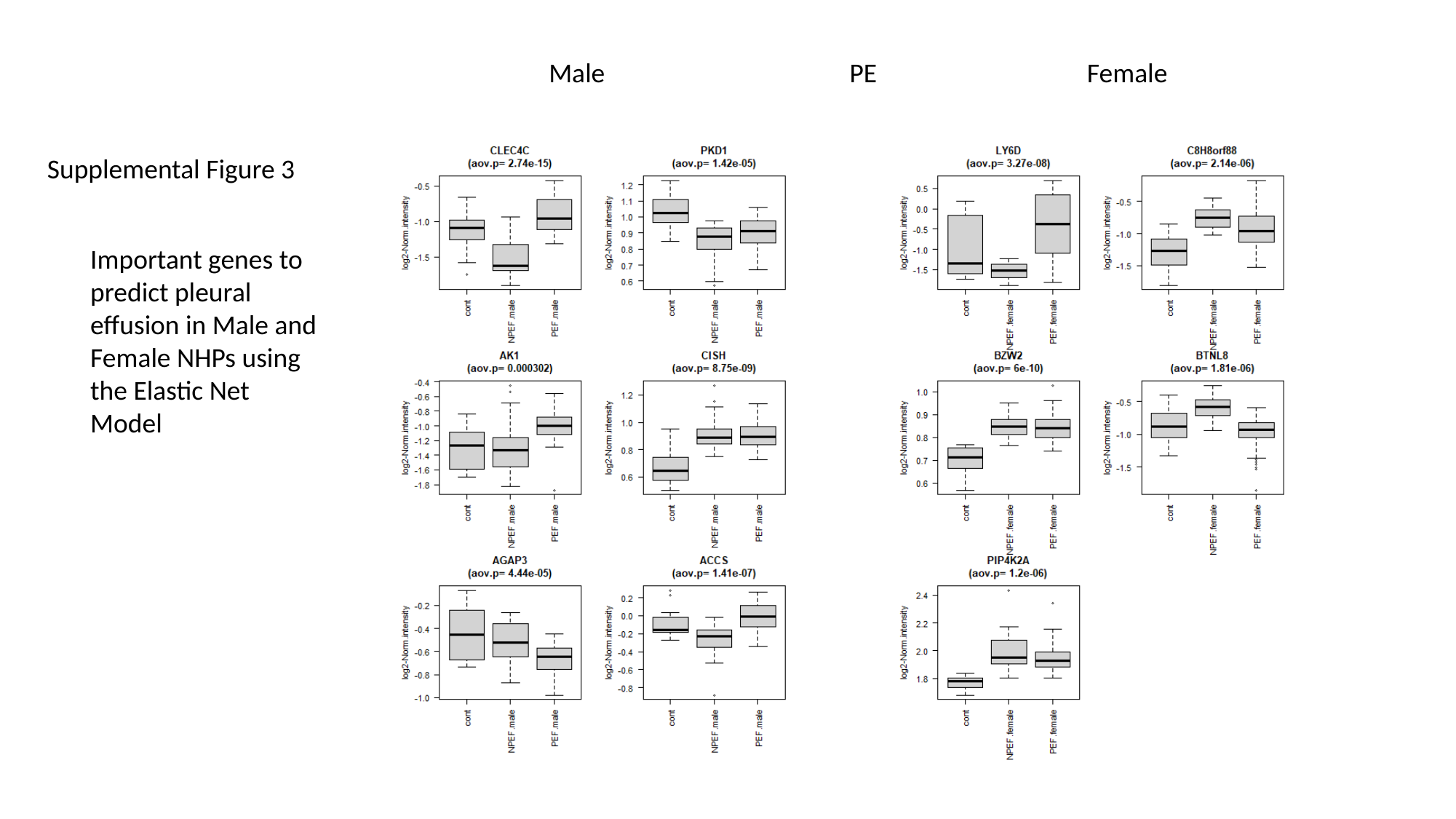

Male
PE
Female
Supplemental Figure 3
Important genes to predict pleural effusion in Male and Female NHPs using the Elastic Net Model
