# Supplementary figures and images for: Whole-blood RNA biomarkers for predicting survival in non-human primates following thoracic radiation
Source: Sci Rep. 2024 Oct 3;14:22957. doi: 10.1038/s41598-024-72975-y (PMC11449919; doi:10.1038/s41598-024-72975-y)

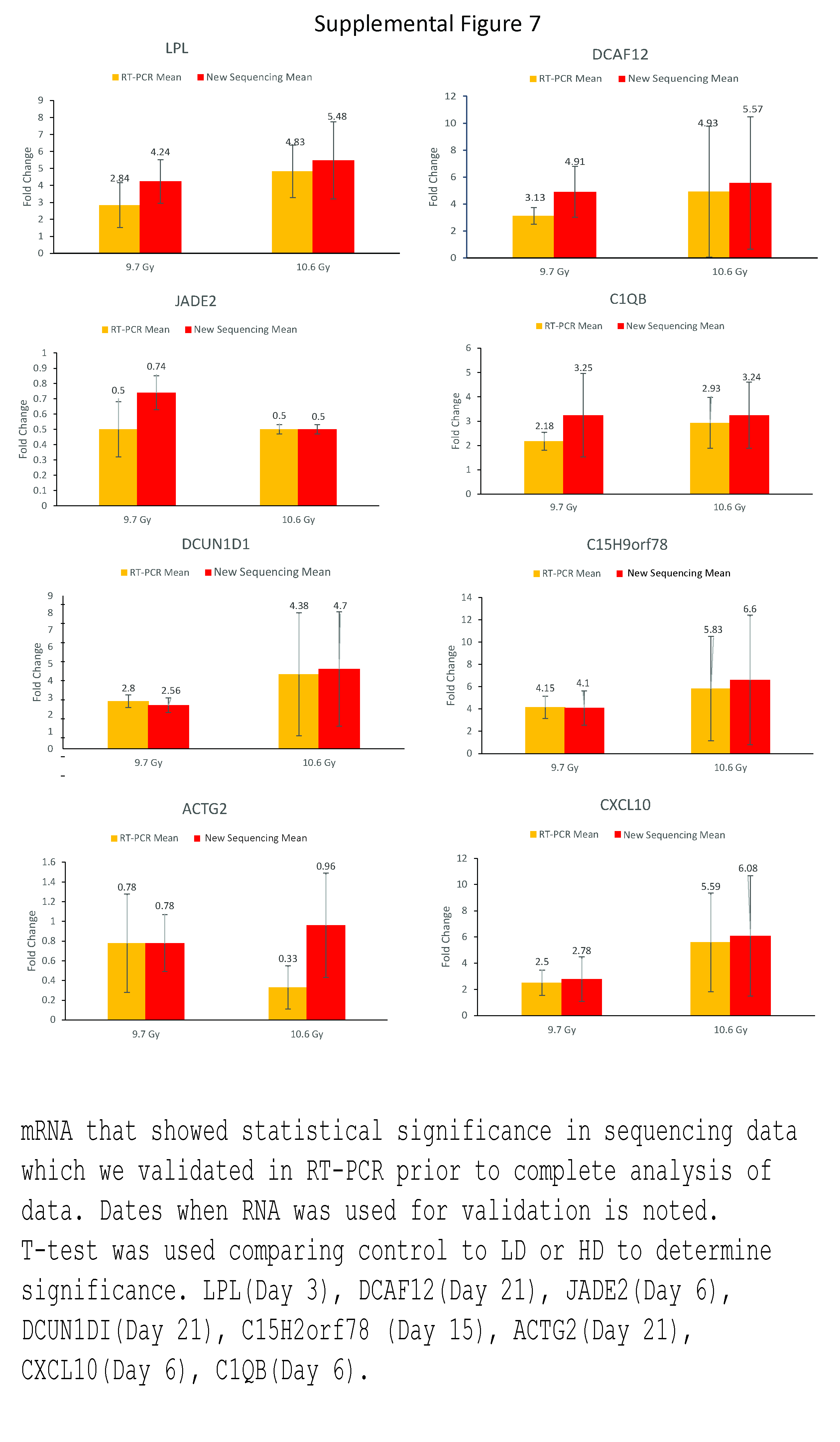

Supplement: Supplementary file 2 — Supplementary Material 2 [file 41598_2024_72975_MOESM2_ESM.tif]
